# Supplementary material for: Evaluation of knowledge and awareness of diabetes in higher secondary level students of Kaski district: A cross-sectional study
Source: PLoS One. 2025 Feb 28;20(2):e0313755. doi: 10.1371/journal.pone.0313755 (PMC11870340; doi:10.1371/journal.pone.0313755)
Supplement: S1 Text — (DOCX) [file pone.0313755.s002.docx]

Questionnaire for the evaluation of knowledge of +2 Science students of ………………..School.

Address:…………………………

**Namaskar,** we would request you to take part in a survey session in which you have to complete a questionnaire. You have the right to withdraw at any point during the study, for any reason, and without any prejudice. All the information provided will be kept confidential and used for research purposes only.

Would you like to participate in the survey?

a. Yes b. No

**Demographic information:**

**Name**: **Age**:

**Gender**: Male/female/other ……. **Class**:

**Assessment of Knowledge of Students**

1. Have you ever heard of diabetes? 🡪 a. Yes b. No
2. What kind of disease is Diabetes?
   1. Communicable (सरुवा रोग हो) b. Non-communicable (सरुवा रोग हैन)
3. Diabetes starts with some defects or problems in an organ in our body. Which is that organ?
   1. Liver b. Heart c. Kidney d. Pancreas
4. Which hormone is associated with diabetes?

a. Insulin b. Growth hormone

c. Testosterone d. Thyroid hormones

1. Diabetes is a condition in which the level of.………………………in blood increases and crosses the normal value.
2. How many types of major Diabetes are there?
   1. 1 b. 2 c. 3. d. 4
3. Which is the most common type of diabetes (affecting greater population)? 🡪

a. Type - I b. Type - II c. both

1. Which age group is more likely to suffer from Type II diabetes?
   1. 10-20 yrs c. 20-35 yrs
   2. More than 35 d. All age groups

### Type II diabetes can be prevented (रोकथाम) It is…. a. True b. False

### Type II diabetes can be delayed (रोग सुरु भइ हाले पनि अलि पछि, धेरै उमेर भए पछि सुरु होस् ) It is…. a. True b. False

1. Do you know any risk factors of diabetes? (जोखिम बढाउने कारक/ तत्वो)

Ans……………………..

1. Diabetic patients may have complications (जटिलता) like blindness.
   1. Yes b. No c. Don’t know
2. Diabetic patients may have complications **(जटिलता)** like Kidney failure.
   1. Yes b. No c. Don’t know
3. What are the common symptoms of diabetes?

a. Excessively thirsty **बढी तिर्खा लाग्नु** b. Excessive urination **पटकपटक** पिसाब लाग्नु

c. Diarrhea d. All of the above

1. Apart from oral medicine, diabetes patients also use injectable drug. Do you know what it is? 🡪 a. Yes, I know. It is ………….. b. No, I don’t know
2. What is the appropriate method for controlling Diabetes :
   1. Medication only
   2. Medication and controlled diet
   3. Medication, controlled diet and regular exercise
3. Diabetic patient should follow a special diet? 🡪 a. Yes b. No
4. The normal fasting blood glucose level is: (fasting – no food for greater than 8 hrs)
   1. Below 100 mg/dL b. 120 mg/dL
      1. 150 mg/dL  d. 200 mg/ dL
5. A random blood glucose level of ……... mg/dL or higher typically means you have diabetes.
   1. 100 mg/dL c. 120 mg/dL
   2. 150 mg/dL d. 200 mg/dL
6. Do you have any diabetic patient in your family?
   1. Yes (Specify the age ………….) b. No
7. An adult person (more than 35 yrs age) has a little higher glucose level but not as high as diabetic patient. What is the appropriate way to prevent diabetes?
   1. Take medicine
   2. Control diet
   3. Control diet and do regular exercise
   4. A and B
8. What kind of food do you need to control if you have diabetes mellitus? मधुमेह रोगीहरुले कस्तो प्रकारका खानामा ध्यान दिनुपर्छ? a._____________________ b. Don’t know
9. How diabetes is diagnosed? मधुमेह भए नभए को जाच्ने तरिका के के छन्?
   1. Fasting blood sugar खाली पेटमा रगत जाच्ने
   2. Post prandial blood sugar खाना खाएपछि रगत जाच्ने
   3. X-Ray
   4. All of above
   5. A and B
10. Choose the correct statement (Can select more than one options)
    1. Early detection of diabetes helps to prevent its complications
    2. Children are at more risk than the elderly people for type II diabetes
    3. Diabetes patients can eat any type of diet
    4. All of the above
11. Do the diabetic patients should visit the eye doctor regularly? 🡪

a. Yes b. No

1. Wound healing takes long time in diabetic patient.🡪 a. Yes b. No
2. Glucose is the building block of
3. Carbohydrate b. Protein c. vitamin d. Fat

Disease-specific information (11)

Symptoms (4)

Treatment (2)

Prevention (3)

Complications (3)
