# Supplementary material for: Evaluation of knowledge and awareness of diabetes in higher secondary level students of Kaski district: A cross-sectional study
Source: PLoS One. 2025 Feb 28;20(2):e0313755. doi: 10.1371/journal.pone.0313755 (PMC11870340; doi:10.1371/journal.pone.0313755)
Supplement: Attachment — Submitted filename: Response to Reviewers.docx [file pone.0313755.s003.docx]

“Response to Reviewers”

Dear Sir/Madam,

We are resubmitting this article again as a new article due to some circumstances which has been explained in our other response letter. The pervious article’s submission number is PONE-D-24-22752.

There was some error regarding the date of data collection in our manuscript. Actually, we mentioned the date of data collection from January 2021 which was before the date that we obtained the ethical approval i.e. September 2022. This was our mistake as we could not correct the date. Actually, we had done some pilot studies from January 2021 to evaluate the questionnaire for the proposal. Those data were not included in the article. We started our research study after obtaining approval i.e. from Sep 2022. Due to this mistake, there was issue of data collection before obtaining ethical approval. That’s why we are resubmitting the same article as a new article according to the guidance of the editor.

Now we have corrected it to eliminate such mistakes in the manuscript. Thank you for your consideration.

Regards,

Ramakanta Lamichhane

Nepal.
